# Regular Practice of Moderate Physical Activity by Older Adults Ameliorates Their Anti-Inflammatory Status

**DOI:** 10.3390/nu10111780

**Published:** 2018-11-16

**Authors:** Miguel D. Ferrer, Xavier Capó, Miquel Martorell, Carla Busquets-Cortés, Cristina Bouzas, Sandra Carreres, David Mateos, Antoni Sureda, Josep A. Tur, Antoni Pons

**Affiliations:** 1 Grup de Nutrició Comunitària i Estrès Oxidatiu, Departament de Biologia Fonamental i Ciències de la Salut, Universitat de les Illes Balears, 07122 Palma, Spain; miguel-david.ferrer@uib.es (M.D.F.); xavier.capo@uib.es (X.C.); martorellpons@gmail.com (M.M.); carla_busquets@hotmail.com (C.B.-C.); cristina.bouzas@uib.es (C.B.); sandra.carreres@uib.cat (S.C.); david-mateos@hotmail.com (D.M.); antoni.sureda@uib.es (A.S.); pep.tur@uib.es (J.A.T.); 2 CIBER: CB12/03/30038 Fisiopatología de la Obesidad la Nutrición, CIBEROBN, Instituto de Salud Carlos III (ISCIII), University of Balearic Islands, 07122 Palma, Spain; 3 Laboratori de Ciències de l’Activitat Física, Departament de Biologia Fonamental i Ciències de la Salut, Universitat de les Illes Balears, 07122 Palma, Spain; 4 Departamento de Nutrición y Dietética, Facultad de Farmacia, Universidad de Concepción, 4070386 Concepción, Chile

**Keywords:** immunity, inflammation, metabolism, physical activity

## Abstract

A chronic inflammatory state is a major characteristic of the aging process, and physical activity is proposed as a key component for healthy aging. Our aim was to evaluate the body composition, hypertension, lipid profile, and inflammatory status of older adults, and these factors’ association with physical activity. A total of 116 elderly volunteers were categorized into terciles of quantitative metabolic equivalents of task (MET). Subjects in the first and third terciles were defined as sedentary and active subjects, respectively. Anthropometric and biochemical parameters, hemograms, and inflammatory markers were measured in plasma or peripheral mononuclear blood cells (PBMCs). The active groups exercised more than their sedentary counterparts. The practice of physical activity was accompanied by lower weight, fat mass, body mass index, and diastolic blood pressure when compared to a more sedentary life-style. Physical activity also lowered the haematocrit and total leukocyte, neutrophil, and lymphocyte counts. The practice of exercise induced a decrease in the IL-6 circulating levels and the TLR2 protein levels in PBMCs, while the expression of the anti-inflammatory IL-10 was activated in active subjects. The regular practice of physical activity exerts beneficial effects on body composition and the anti-inflammatory status of old people.

## 1. Introduction

Ageing is an unavoidable process in all animals and is characterized by progressive accumulation of cell and organ damage, which result in organism malfunction. In the past few centuries, the proportion of elderly people has been continuously increasing worldwide and is projected to reach 19.3% of total population by 2050 [1]. Although ageing has an unavoidable and intrinsic component, it is also importantly modulated by several external factors, such as exposure to chemicals, lifestyle, or nutrition [2]. Therefore, it seems clear that the progression of ageing can be, at least in part, counteracted by the combination of adequate nutritional intake and a healthy lifestyle, the latter including the regular practice of physical activity [3].

Several studies have evidenced the beneficial effects of physical activity on longevity, showing that regular physical activity is associated with a 30% reduction in the risk of mortality in subjects without CV disease [4], which might correspond to one to two years of additional life [5]. On the other hand, physical inactivity causes 6–10% of the burden of several diseases (including coronary heart disease, diabetes, and cancer) and 9% of premature mortality [6]. In fact, regular physical activity prescription for healthy ageing is a key point for chronic disease management and prevention [4,7]. These positive effects of physical activity might be related to greater conservation of lean tissue [8], lower body mass, and less relative body fat [9] in old adults engaging in high levels of physical activity in comparison to individuals who are more sedentary.

Regular physical activity has also been shown to reduce the risk of several diseases, such as cardiovascular disease, stroke, hypertension, type 2 diabetes, osteoporosis, obesity, colon cancer, breast cancer, anxiety, and depression [10]. Most of these diseases are directly or indirectly related to inflammation processes. In this instance, the benefits of exercise on life-span have been related to different cardioprotective mechanisms, including effects on endothelial function and inflammation [4]. Actually, it has been shown that exercise training exerts anti-inflammatory effects in aged or diseased populations [11], and these effects might be mediated by decreases in TNF-α expression in skeletal muscle, among other effectors [12]. Increased risk of chronic diseases has been associated with elevated inflammation markers [13,14], while the practice of physical activity has shown to reduce pro-inflammatory biomarkers such as C-reactive protein [15], TNF-α [16], or interleukin (IL) 6 [17,18]. 

Old people usually face a situation of chronic low-grade inflammation. It has been stated that inflammatory cytokines are elevated, and anti-inflammatory cytokine concentrations are lowered, in healthy adults over 50 years of age [19]. This behaviour has been associated with redistribution of body fat and concomitant increases in circulating fatty acids that lead to the activation of proinflammatory macrophages [20]. This chronic low-grade inflammatory status, termed inflamm-aging by some authors [14,21], appears to be a major component of the most common age-related diseases, such as diabetes, osteoporosis, cardiovascular diseases, and cancer. 

Therefore, the aim of this study was to evaluate the body composition, hypertension, and lipid metabolic profile, as well as the inflammatory status, of older adults, as well as its association with the regular practice of physical activity.

## 2. Materials and Methods

### 2.1. Subjects and Study Design 

A total of 116 elderly volunteers (58 men aged between 55 and 80 years and 58 women aged between 60 and 80 years) participated in the study. These volunteers were selected from a larger study population conforming the PHYSMED project (with a total of 380 participants), a multi-centre, cross-sectional study aiming at identifying cardiovascular risk factors in sedentary and active elderly subjects. The 116 volunteers included in this study were recruited in social and municipal clubs, health centres, and sport clubs in different villages and cities of Mallorca, Spain. Exclusion criteria included being institutionalized, suffering from a physical or mental illness that would have limited their participation in physical fitness or their ability to respond to questionnaires, chronic alcoholism or drug addiction, and intake of drugs for clinical research over the past year.

The physical activity performed by the participants was measured using the Minnesota Leisure-time Physical Activity Questionnaire previously validated for the Spanish old adult population [22,23]. This questionnaire included a list of physical activities, and the participants were asked about what type of leisure-time physical activities (LTPA) they had performed during the last year. The participants estimated the duration of the activities performed each hour/week by using metabolic equivalents of task (MET, defined as 1 kcal/kg/hour and equivalent to the energy cost of sitting quietly) [24]. The resulting quantitative MET for each participant were categorized into terciles [25], and subjects in the first and the third terciles were selected to take part in this study and defined as sedentary and active subjects, respectively. 

The study was conducted according to the guidelines laid down in the Declaration of Helsinki, and all procedures were approved by the Ethics Committee of Clinical Research of the Balearic Islands (CEIC-IB, ref. 1295/09 PI). All the subjects were informed of the purpose and demands of the study before giving their written consent to participate.

Venous blood samples were obtained from the antecubital vein of participants in resting conditions after overnight fasting. The peripheral blood mononuclear cell (PBMC) fraction was purified from whole blood following an adaptation of the method described by Boyum [26] using Ficoll-Paque PLUS reagent (GE Healthcare). This procedure ensures a PBMC purity and viability of 95 ± 5%.

### 2.2. Anthropometric Characteristics

Anthropometric measurements were performed by well-trained dieticians who underwent identical and rigorous training as an effort to minimize the effects of inter-observer variation. Height was determined using a mobile anthropometer (Seca 213, SECA Deutchland, Hamburg, Germany) to the nearest millimetre, with the subject’s head in the Frankfurt plane. Body weight, body fat, and muscle mass were determined using a Segmental Body Composition Analyzer (Tanita BC-418, Tanita, Tokyo, Japan). The participants were weighed in bare feet and light clothes, subtracting 0.6 g for their clothes. Body mass index (BMI) was calculated using the following formula: BMI = mass (kg)/squared height (m). 

### 2.3. Biochemical Parameters and Hemogram

Glucose, triglycerides, total, high-density lipoprotein (HDL), low-density lipoprotein (LDL) and very low-density lipoprotein (VLDL) cholesterol, urea, uric acid, and creatinine were determined by standard procedures using commercial clinical kits in an autoanalyzer system (Technicon DAX System).

Haematological parameters and hemogram were determined in an automatic flow cytometer analyser Technicon H2 (Bayer, Leverkusen, Germany) VCS system. Haemoglobin concentration was determined using Drabkin reagent (Sigma Aldrich, St. Louis, MO, USA).

### 2.4. Circulating Inflammatory Parameters

IL-6, sCD62L, and sICAM3 plasma levels were determined using individual ELISA kits from Diaclone (Besançon, France). TNFα was determined using the RayBiotech (Norcross, GA, USA) ELISA kit. All procedures were performed following the supplier instructions for use.

MPO activity in plasma was measured by guaiacol oxidation, under identical conditions to those previously described [27].

### 2.5. mRNA Gene Expression

mRNA expressions were determined by real time-polymerase chain reaction (RT-PCR). For this purpose, mRNA was isolated from PBMC by extraction with Tripure Isolation Reagent (Roche, Basel, Switzerland). cDNA was synthesized from 1 µg total RNA using reverse transcriptase with oligo-dT primers. Quantitative PCR was performed using the LightCycler instrument (Roche Diagnostics, Basel, Switzerland) with DNA-master SYBR Green I. The primers used are shown in Table 1. For all PCRs, there was one cycle at 95°C for 10 min, followed by 40 cycles at the conditions shown in Table 1.

### 2.6. Western Blot Analysis in PBMCs

Toll-Like Receptor (TLR) 2 and 4 protein levels were determined in PBMCs by Western blot. Protein extracts were analysed by SDS–polyacrylamide gel electrophoresis (SDS–PAGE). Total protein concentrations were measured by the method of Bradford [28]. 80 μg of total protein was loaded on a 12% agarose gel. Following electrophoresis, samples were transferred onto a nitrocellulose membrane and incubated with a primary monoclonal anti-TLR2 or anti-TLR4 antibody (Santa Cruz Biotechnology, Dallas, TX, USA) and a secondary anti-mouse IgG peroxidase-conjugated antibody. Protein bands were visualized by Immun-Star^®^ Western C^®^ Kit reagent (Bio-Rad Laboratories, Hercules, CA, USA) Western blotting detection systems. The chemiluminiscence signal was captured with a Chemidoc XRS densitometer (Bio-Rad Laboratories) and analyzed with Quantity One-1D Software (Bio-Rad Laboratories). 

### 2.7. Statistical Analysis

Statistical analysis was carried out using a statistical package for social sciences (SPSS 22 for Windows, SPSS Inc., Chicago, IL, USA). Results are expressed as mean ± standard error of the mean (SEM) and *p* < 0.05 was considered statistically significant. The statistical significance of the data was assessed by a two-way analysis of variance (ANOVA). The statistical factors analysed were (S) sex and (E) exercise. When significant effects were found, one-way ANOVA was used to determine the differences between the groups involved.

## 3. Results

The anthropometric characteristics of the participants are shown in Table 2. The active groups (both male and female) exercised more than their sedentary counterparts, as evidenced by the significantly higher degree of physical activity measured in MET-hours/week. No differences in the degree of physical activity performed were evidenced between males and females. However, males were taller, weighed more, and presented higher fat-free mass and body mass index than females. The practice of regular physical activity was accompanied by significantly lower total weight, fat mass, body mass index, and diastolic blood pressure when compared to a more sedentary life-style.

Neither sex or exercise influenced glucose or triglyceride circulating levels (Table 3). Total circulating cholesterol was, however, significantly affected by the sex of the participants, with higher levels observed in women when compared to men. These higher levels of total cholesterol found in females seem attributable to higher HDL-cholesterol levels, which were also higher in women compared to men. HDL-cholesterol circulating levels were also significantly affected by exercise, with those groups of active participants presenting higher levels than their sedentary counterparts. A significant effect of sex was also evidenced in the circulating levels of uric acid and creatinine: females presented significantly lower levels of uric acid and creatinine than their respective male counterparts.

Sex also affected several hemogram parameters, as shown in Table 3. In this instance, females presented significantly lower counts of red blood cells (which resulted in a lower haematocrit and lower haemoglobin content) and eosinophils, as well as a higher platelet count. On the other side, the practice of physical activity lowered the haematocrit, through the significant decrease on total leukocyte count, as well as neutrophil and lymphocyte count.

The circulating levels of key pro-inflammatory proteins are shown in Table 4. No effects of sex were evidenced in any of the circulating pro-inflammatory proteins measured. A significant effect of exercise was observed only in IL-6 levels. The practice of exercise induced a decrease in the circulating levels of IL-6, although this decrease was only significant in the group of females.

**Figure 1:**
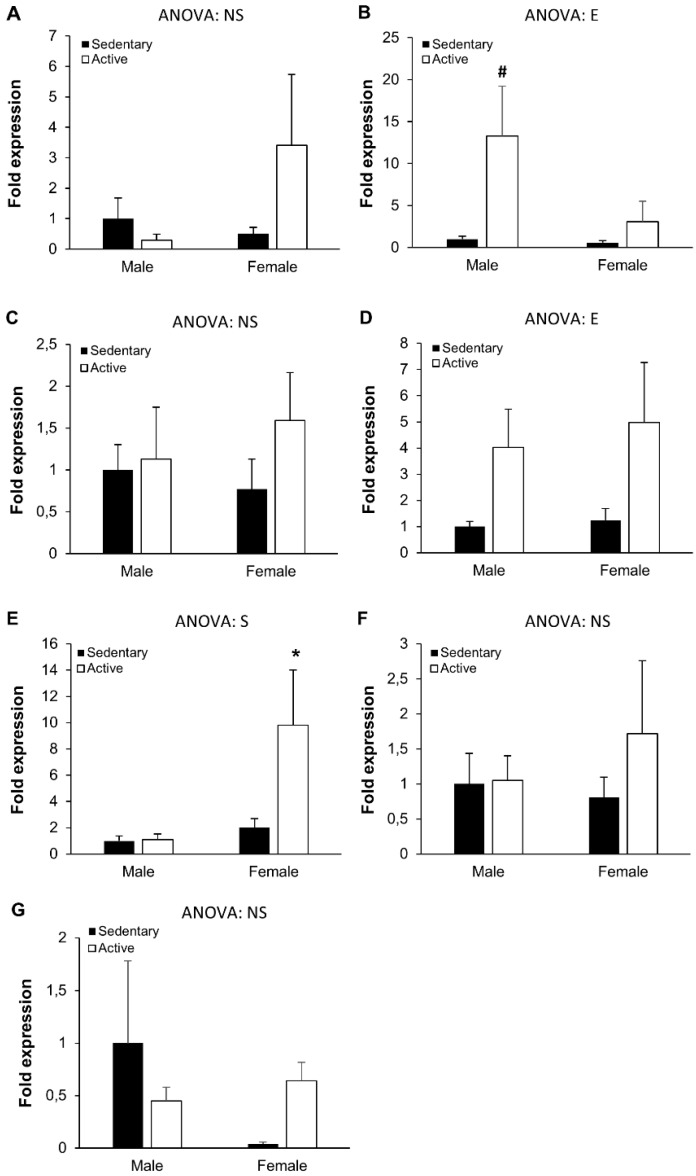
Peripheral blood mononuclear cells gene expression. (**A**) Interleukin(IL)-1 receptor antagonist, (**B**) IL-10, (**C**) IL-1β, (**D**) NF-κB, (**E**) TLR4, (**F**) TNFα, (**G**) IL-6. Results represent mean ± SEM. Statistical analysis: two-way ANOVA, *p* < 0.05. (S) effect of sex, (E) effect of exercise, (NS) non-significant. (*) Significant differences between sexes, (#) significant differences between sedentary and active groups.

**Figure 2:**
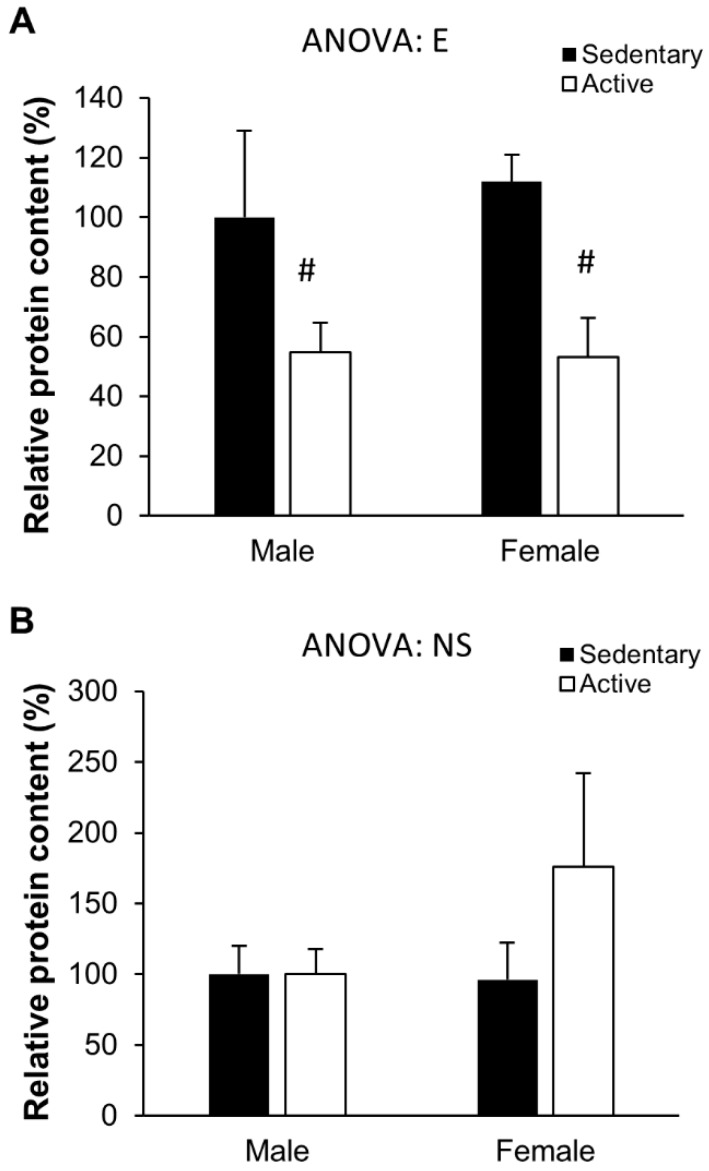
Peripheral blood mononuclear cells protein levels. (**A**) Tol-like receptor (TLR)2 and (**B**) TLR4. Results represent mean ± SEM. Statistical analysis: two-way ANOVA, *p* < 0.05. (E) effect of exercise. (#) Significant differences between sedentary and active groups.

The inflammatory status of the organism was additionally studied through the gene (Figure 1) and protein (Figure 2) expression of pro- and anti-inflammatory cytokines in PBMC. The regular practice of physical activity influenced the expression of the anti-inflammatory IL-10, with significantly higher expression levels in active males compared to sedentary males. A similar pattern of response, although non-significant, was also observed in females. Similarly, exercise also significantly influenced the gene expression of NF-κB, tending to higher expressions in active participants when compared to their sedentary counterparts. A significant effect of sex was observed regarding TLR4 gene expression: significantly higher expression of this gene was observed in active females when compared to active males. 

A similar (but non-significant) tendency was also observed in TLR4 protein levels (Figure 2). Finally, TLR2 protein levels were affected by exercise, as evidenced by the significantly lower TLR2 protein levels in the PBMC of active vs sedentary participants (both in males and females). 

## 4. Discussion

Aging has been associated with the functioning of the immune system, and more concretely with inflammatory responses. A chronic, low-grade inflammatory state, called inflamm-aging by some authors [14,21], has been proposed as being responsible for a progressive pro-inflammatory status, which appears to be a major characteristic of the aging process and age-related disease [29]. Therefore, the modulation of the inflammatory status throughout one’s life might be an adequate strategy to attain healthy ageing. In this instance, the practice of physical activity has been proposed as a key component of healthy aging [4,30], and the benefits exerted by exercise might be attributable to the acquisition of an anti-inflammatory status. In the present study, we demonstrate that regular practice of physical activity exerts beneficial effects on body composition and the anti-inflammatory status of old people. 

The physical activity performed by the participants was measured in the current study using the Minnesota Leisure-time Physical Activity Questionnaire, which had been previously validated for the Spanish old adult population [22,23], and the participants estimated the duration of the activities performed in hour/week by using metabolic equivalents of task (MET, defined as 1 kcal/kg/hour and equivalent to the energy cost of sitting quietly) [24]. The subjects in the first tercile (<82 MET-hours/week) were defined as sedentary, while the subjects in the third tercile (>84 MET-hours/week) were defined as active subjects. As expected by this classification, the active subjects performed around three-fold more physical activity than the sedentary subjects, both in the male and female groups. This regular practice of physical activity translated into a lower weight, a lower fat mass content, and a lower BMI. These results are in accordance with previous reports that evidenced that the regular practice of physical activity by old people reduces fat mass and BMI and increases fat-free mass [31,32]. These effects on body composition were also accompanied by reduced diastolic blood pressure in the physically active subjects, as has been extensively reported in subjects performing aerobic exercise [33]. The effects of sex on the body composition were also evidenced, as women presented lower fat-free mass and higher fat mass than their male counterparts. 

The practice of physical activity also had positive effects on the levels of HDL-cholesterol, which is in accordance with previous reports [34,35]. Lipid parameters were also affected by sex, with women presenting higher total cholesterol circulating levels, which were attributable to higher HDL-cholesterol. These results are in accordance with previous reports on different European populations showing that HDL-cholesterol circulating levels are higher in women than in men [36,37].

The active subjects presented a lower haematocrit than their sedentary counterparts, but similar values of red blood cells counts, haemoglobin levels, and mean corpuscular erythrocyte volume. The lower haematocrit was accompanied by a certain degree of leucopoenia. Although decreases in the number of circulant erythrocytes have been reported in response to acute bouts of physical activity [38], these changes are not always found in well-trained subjects [39], and even increases in the haematocrit and erythrocyte number have been reported in both amateur and professional sportsmen after maximal and submaximal tests and a cycling stage [40]. The leucopoenia found in the active participants of the current study was explained by lower counts of both neutrophils and lymphocytes and is in accordance with previous studies reporting a certain degree of leucopoenia in response to the regular practice of physical activity, which in turn is interpreted as part of an anti-inflammatory response [41]. 

The systemic inflammatory status of the participants in the study was evaluated through the measurement of circulating pro- and anti-inflammatory proteins and gene and protein expression of different cytokines in PBMCs. A decrease in the circulating levels of IL6 was observed in the active groups. Although IL6 can also exert anti-inflammatory activity (after an acute bout of exercise, IL-6 may induce the anti-inflammatory cytokines IL-10 and IL-1ra [42]), the presence of chronic circulating concentrations of IL6 can induce an acute phase immune response [43], and the regular practice of physical activity induces lower basal concentrations of IL-6 when compared to a sedentary lifestyle [44]. The plasma concentration of this interleukin has been associated with lower muscle mass [45] and higher adiposity [46], although in the present study we have not evidenced differences in fat-free mass. Although a reduction in IL6 levels was observed in the group of active volunteers, no changes in circulating TNF-α were evidenced. Gene expression of pro-inflammatory cytokines such as IL1β or TNF-α or the pro-inflammatory receptor TLR4 in PBMCs were not significantly affected by the practice of regular exercise. These results are in accordance with previous studies reporting that IL1β does not respond to different degrees of exercise, including low intensity aerobic exercise, high intensity aerobic exercise, or a combination of high intensity aerobic and resistance exercise [34]. Although no effects of physical activity were observed either on the gene expression of IL1ra, a significant activation of the anti-inflammatory cytokine IL10 gene expression was observed. Higher levels of IL10 in response to physical activity have been previously reported [34,35], and these increases have been actually related to a decrease in fat mass. In fact, the chronic inflammatory state has been related to the adiposity, and the influence of physical activity in body composition may therefore influence the inflammatory state [31,45,47,48]. The fact that anti-inflammatory cytokine concentrations are lowered in healthy adults over 50 years of age has also been associated with redistribution of body fat [20]. Our current results (lower fat mass and higher IL10 expression in active subjects) are in accordance with previous data and reinforce the anti-inflammatory effect of the regular practice of physical activity in old people. 

A significant overexpression of the transcription factor NFκB was also observed in the physically active groups. This nuclear factor can be activated through the action of pro-inflammatory cytokines (such as TNFα), but it can also be activated by ROS and/or RNS [49]. Once activated, the nuclear factor migrates to the nucleus and may induce the expression of a wide variety of genes, including inflammatory cytokines such as TNFα, IL-6, and IL-1β [50,51], but also antioxidant enzymes such as superoxide dismutase and nitric oxide synthase [52,53]. As the PBMC gene expression profile shows no evidence of a pro-inflammatory phenotype, we might interpret the activation of NFκB through the ROS/RNS route rather than a proinflammatory response. In this instance, the regular practice of physical activity exposes the organism to a sustained and continuous production of low levels of ROS, and these low levels of ROS have been shown to act as second messengers leading an antioxidant and anti-inflammatory response through the activation of NFκB and other genes [52,53].

The protein levels of the inflammation-related receptors TLR2 and TLR4 were also measured. While no effects were observed regarding TLR4 levels, a decrease in both sexes in the protein levels of TLR2 was observed. Activation of the TLR4 signalling pathway stimulates an increase in pro-inflammatory cytokines such as TNFα, IL1β, or IL6, and it has been previously described that physical activity may downregulate TLR4 expression in the immune cells [54,55], together with downstream cytokines such as TNFα [16], IL1β [34], and IL6 [18]. However, a recent study reported that neither TLR4 nor TNFα responded to resistance training with or without weight loss [32], which is in accordance with our own results. Although TLR4 is usually more sensitive TLR in response to physical activity, we observed a down-regulation of TLR2 in active subjects but not of TLR4. TLR2 is another member of the TLRs family that is also involved in the cell response to immune stimuli, and shares with TLR4 its downstream signalling cascade. A recent systematic review showed that chronic exercise has anti-inflammatory effects on the organism through the downregulation of both TLR2 and TLR4 at the protein and gene expression levels [56], which is in accordance with our results. 

Taken together, our results show that the regular practice of physical activity by older adults ameliorates their anthropometric characteristics by reducing their weight, fat mass, and body mass index. This effect in their body composition is accompanied by a healthier status, with lower diastolic blood pressure and higher levels of circulating HDL-cholesterol. The changes in the fat body composition and lipid profile might be responsible for the observed attenuation of pro-inflammatory parameters, such as the reduced count of lymphocytes and neutrophils, reduced IL6 circulating levels, and the changes in the expression of pro- and anti-inflammatory proteins in PMBCs. In conclusion, the regular practice of physical activity (> 84 MET-hours/week) by older adults ameliorates their anti-inflammatory status. 

## Figures and Tables

**Table 1:** Primers and conditions used in the PCRs.

Gene	Primer		Conditions	
18S	Fw:	5′-ATG TGA AGT CAC TGT GCC AG-3′	95 °C 60 °C 72 °C	10 s 10 s 12 s
	Rv:	5′-GTG TAA TCC GTC TCC ACA GA-3′		
IL-1ra	Fw:	5′-GAA GAT GTG CCT GTC CTG TGT-3′	95 °C 60 °C 72 °C	10 s 10 s 15 s
	Rv:	5′-CGC TCA GGT CAG TGA TGT TAA-3′		
IL10	Fw:	5′-AGA ACC TGA AGA CCC TCA GGC-3′	95 °C 60 °C 72 °C	10 s 10 s 15 s
	Rv:	5′-CCA CGG CCT TGC TCT TGT T-3′		
IL1β	Fw:	5′-GGA CAG GAT ATG GAG CAA CA-3′	95 °C 58 °C 72 °C	10 s 10 s 15 s
	Rv:	5′-GGC AGA CTC AAA TTC CAG CT-3′		
NFκB	Fw:	5′-AAA CAC TGT GAG GAT GGG ATC TG-3′	95 °C 60 °C 72 °C	10 s 10 s 15 s
	Rv:	5′-CGA AGC CGA CCA CCA TGT-3′		
TLR4	Fw:	5′-GGT CAC CTT TTC TTG ATT CCA-3′	95 °C 60 °C 72 °C	10 s 10 s 15 s
	Rv:	5′-TCA GAG GTC CAT CAA ACA TCA C-3′		
TNFα	Fw:	5′-CCC AGG CAG TCA GAT CAT CTT CTC GGA A-3′	94 °C 63 °C 72 °C	10 s 10 s 15 s
	Rv:	5′-CTG GTT ATC TCT CAG CTC CAC GCC ATT-3′		
IL6	Fw:	5′-ACC TGA ACC TTC CAA AGA TGG C-3′	95 °C 63 °C 72 °C	10 s 10 s 15 s
	Rv:	5′-TCA CCA GGC AAG TCT CCT CAT TG-3′		

The relative quantification was performed by standard calculations considering 2^(−ΔΔ*C*t)^. mRNA levels of sedentary males were arbitrarily referred to as 1. The expression of the target gene was normalized with respect to ribosomal 18S.

**Table 2:** Anthropometric characteristics of the participants.

		Sedentary	Active	ANOVA		
				Sex	Exercise	SxE
Age (years)	Male	64.6 ± 1.1	62.5 ± 0.9	0.000	0.339	0.281
	Female	67.3 ± 1.1	67.4 ± 1.0 *			
Physical activity (MET-hours/week)	Male	40.4 ± 4.4	141 ± 9 #	0.602	0.000	0.071
	Female	48.4 ± 3.3	126 ± 6 #			
Weight (kg)	Male	86.1 ± 1.9	78.2 ± 2.0 #	0.000	0.000	0.875
	Female	69.3 ± 2.2 *	62.0 ± 1.7 *			
Height (cm)	Male	170 ± 1	171 ± 1	0.000	0.808	0.624
	Female	157 ± 1 *	156 ± 1 *			
Fat-free mass (kg)	Male	61.1 ± 1.1	58.8 ± 1.4	0.000	0.142	0.531
	Female	41.8 ± 0.9 *	40.9 ± 0.6 *			
Fat mass (kg)	Male	25.0 ± 1.1	19.4 ± 0.9 #	0.090	0.000	0.765
	Female	27.5 ± 1.6	21.2 ± 1.2 #			
Body Mass Index (kg/m^2^)	Male	29.6 ± 0.6	26.8 ± 0.5 #	0.038	0.000	0.874
	Female	28.1 ± 0.8	25.5 ± 0.7 #			
Systolic blood pressure (mm Hg)	Male	141 ± 3	138 ± 4	0.312	0.312	0.796
	Female	138 ± 4	133 ± 3			
Diastolic blood pressure (mm Hg)	Male	84.8 ± 1.4	81.2 ± 1.8	0.099	0.039	0.883
	Female	82.0 ± 2.2	77.8 ± 1.8			

Mean ± SEM. Statistical analysis: two-way ANOVA, *p* < 0.05. (S) effect of sex, (E) effect of exercise, and (SxE) interaction between the two factors. (*) significant differences between sexes; (#) significant differences between sedentary and active groups.

**Table 3:** Biochemical parameters and hemogram of the participants.

		Sedentary	Active	ANOVA		
				Sex	Exercise	SxE
Glucose (mg/dL)	Male	100 ± 2	98.8 ± 2.8	0.636	0.153	0.217
	Female	105 ± 12	87.8 ± 1.8			
Triglycerides (mg/dL)	Male	111 ± 7	100 ± 6	0.360	0.564	0.289
	Female	97.2 ± 6.3	100 ± 6			
Total cholesterol (mg/dL)	Male	197 ± 5	199 + 5	0.016	0.919	0.732
	Female	214 ± 7	211 ± 6			
HDL (mg/dL)	Male	44.7 ± 1.6	51.6 ± 2.2	0.000	0.011	0.520
	Female	57.2 ± 2.0 *	61.3 ± 2.5 *			
LDL (mg/dL)	Male	130 ± 5	127 ± 5	0.360	0.317	0.676
	Female	137 ± 6	130 ± 5			
VLDL (mg/dL)	Male	22.3 ± 1.4	19.9 ± 1.3	0.364	0.532	0.285
	Female	19.5 ± 1.3	20.1 ± 1.8			
Urea (mg/dL)	Male	36.3 ± 1.5	36.2 ± 1.7	0.754	0.883	0.909
	Female	36.0 ± 1.5	35.6 ± 1.2			
Uric acid (mg/dL)	Male	6.25 ± 0.21	6.03 ± 0.18	0.000	0.087	0.515
	Female	5.04 ± 0.22 *	4.56 ± 0.20 *			
Creatinine (mg/dL)	Male	0.829 ± 0.018	0.841 ± 0.016	0.000	0.978	0.460
	Female	0.728 ± 0.013 *	0.716 ± 0.018 *			
Red blood cells (10^6^/mm^3^)	Male	5.03 ± 0.08	4.90 ± 0.07	0.000	0.103	0.860
	Female	4.62 ± 0.06 *	4.52 ± 0.08 *			
Haemoglobin (g/dL)	Male	15.5 ± 0.2	15.3 ± 0.2	0.000	0.056	0.798
	Female	14.2 ± 0.2 *	13.8 ± 0.1 *			
Haematocrit (%)	Male	46.0 ± 0.5	45.0 ± 0.6	0.000	0.048	0.971
	Female	42.1 ± 0.5 *	41.1 ± 0.4 *			
Mean corpuscular volume (fL)	Male	91.7 ± 0.9	91.9 ± 0.6	0.480	0.916	0.857
	Female	91.3 ± 0.7	91.2 ± 0.9			
Platelets (10^3^/mm^3^)	Male	222 ± 10	214 ± 8	0.018	0.195	0.743
	Female	246 ± 9	232 ± 8			
Leucocytes (10^3^/mm^3^)	Male	6.39 ± 0.29	5.85 ± 0.21	0.423	0.002	0.244
	Female	6.49 ± 0.32	5.33 ± 0.21 #			
Neutrophils (10^3^/mm^3^)	Male	3.43 ± 0.21	3.15 ± 0.18	0.074	0.006	0.185
	Female	3.35 ± 0.22	2.56 ± 0.13 #			
Lymphocytes (10^3^/mm^3^)	Male	2.20 ± 0.13	1.94 ± 0.09	0.084	0.039	0.997
	Female	2.41 ± 0.14	2.15 ± 0.12			
Monocytes (10^3^/mm^3^)	Male	0.512 ± 0.025	0.526 ± 0.027	0.114	0.306	0.116
	Female	0.511 ± 0.024	0.446 ± 0.022			
Eosinophils (10^3^/mm^3^)	Male	0.220 ± 0.025	0.196 ± 0.020	0.037	0.088	0.586
	Female	0.187 ± 0.023	0.140 ± 0.013			
Basophils (10^3^/mm^3^)	Male	0.035 ± 0.004	0.037 ± 0.004	0.721	0.855	0.775
	Female	0.034 ± 0.004	0.034 ± 0.003			

Mean ± SEM. Statistical analysis: two-way ANOVA, *p* < 0.05. (S) effect of sex, (E) effect of exercise, and (SxE) interaction between the two factors. (*) significant differences between sexes; (#) significant differences between sedentary and active groups.

**Table 4:** Plasma markers of inflammation.

		Sedentary	Active	ANOVA		
				Sex	Exercise	SxE
IL6 (pg/mL)	Male	3.33 ± 0.27	2.55 ± 0.18	0.599	0.001	0.478
	Female	3.39 ± 0.31	2.19 ± 0.35 #			
TNFα (pg/mL)	Male	26.6 ± 3.0	21.5 ± 1.8	0.480	0.148	0.997
	Female	29.1 ± 3.2	23.9 ± 5.6			
sCD62L (ng/mL)	Male	1507 ± 61	1298 ± 77	0.584	0.132	0.825
	Female	1631 ± 239	1351 ± 110			
sICAM3 (ng/mL)	Male	523 ± 24	496 ± 18	0.549	0.318	0.832
	Female	531 ± 20	514 ± 25			
Myeloperoxidase (µkat/mL)	Male	139 ± 43	179 ± 45	0.155	0.799	0.514
	Female	105 ± 36	87 ± 29			

Mean ± SEM. Statistical analysis: two-way ANOVA, *p* < 0.05. (S) effect of sex, (E) effect of exercise, and (SxE) interaction between the two factors. (#) significant differences between sedentary and active groups.
